# Signal integration challenges in African epidemic intelligence: Lessons from the 2026 Bundibugyo virus disease outbreak and application of the PREIS framework

**DOI:** 10.4102/jphia.v17i1.2079

**Published:** 2026-08-27

**Authors:** Hyacinthe R. Zabré

**Affiliations:** 1 Data Science and Informatics Unit, Surveillance and Disease Intelligence Division, Africa Centres for Disease Control and Prevention, Addis Ababa, Ethiopia

**Keywords:** epidemic intelligence, Bundibugyo virus disease, Ebola disease, Public Health Emergency of International Concern, Africa, event-based surveillance, signal integration, reproducible analytics

## Abstract

On 17 May 2026, the Director-General of the World Health Organization determined that Ebola disease caused by Bundibugyo virus in the Democratic Republic of the Congo (DRC) and Uganda constituted a Public Health Emergency of International Concern. Ten days separated the initial alert (05 May) from laboratory confirmation (15 May) although multiple non-laboratory signals were already active. Using this outbreak, we argue that Africa’s principal detection challenge is often not signal availability but the timely interpretive integration of concurrent multi-source signals, and we outline how a reproducible analytical layer – the Pan-African Real-time Epidemiological Intelligence System (PREIS) framework – might support it.

## Background and public health significance

On 05 May 2026, the World Health Organization (WHO) was alerted to a high-mortality outbreak of unknown illness in Mongbwalu Health Zone, Ituri Province, Democratic Republic of the Congo (DRC), including deaths among health workers.^1^ The *Institut national de recherche biomédicale* confirmed Bundibugyo virus disease in 8 of 13 samples on 15 May 2026, and the country declared its 17th Ebola disease outbreak; the same day, Uganda confirmed an imported case in a Congolese traveller who died in Kampala.^1^ On 17 May 2026, the WHO Director-General determined that the event constituted a Public Health Emergency of International Concern (PHEIC).^2^ As of 16 May 2026, WHO reported eight laboratory-confirmed cases, 246 suspected cases and 80 suspected deaths across three Ituri health zones (Bunia, Rwampara and Mongbwalu), plus two imported laboratory-confirmed cases (one fatal) in Kampala.^1,2^ As of 01 July 2026, the DRC outbreak had reached 1460 confirmed cases and 452 deaths, with cases reported across Ituri, North Kivu and South Kivu and continued cross-border risk involving Uganda.^3^

The 10-day interval between the initial alert (05 May) and laboratory confirmation (15 May) is an important operational anchor. During it, multiple signal streams were already active: Syndromic surge reports from Mongbwalu, clusters of health-worker deaths, cross-border mobility along the DRC–Uganda corridor and community mortality patterns.^1^ Existing platforms captured many of these signals – the WHO Epidemic Intelligence from Open Sources (EIOS) initiative,^4^ the WHO African Region Integrated Disease Surveillance and Response (IDSR) framework, whose third edition formally integrates event-based surveillance,^5^ the Africa Centres for Disease Control and Prevention (Africa CDC) event-based surveillance framework,^6^ ProMED-mail,^7^ HealthMap^8^ and the WHO Early Warning, Alert and Response System (EWARS).^9^ The challenge was not signal capture but the interpretive integration of concurrent multi-source signals into a unified detection-decision workflow.

In this article, a signal denotes any data element from a heterogeneous source – laboratory, event-based, community, cross-border or open-source – potentially indicative of an emerging public health event prior to formal case classification. Signal integration failure denotes the systematic non-convergence of concurrent multi-source signals into a unified detection-decision workflow, resulting in delayed epidemic recognition despite pre-existing data. Both definitions are consistent with the WHO glossary for epidemic intelligence,^4^ the Africa CDC event-based surveillance framework^6^ and the companion methodological preprint.^10^

## The Pan-African Real-time Epidemiological Intelligence System framework: Brief description

The Pan-African Real-time Epidemiological Intelligence System (PREIS) is a proposed methodological framework for reproducible epidemic intelligence workflows, designed to organise heterogeneous permitted data streams into standardised, auditable and reviewable intelligence products. Its full description – five-layer architecture, six design principles, a 16-field minimum data model, an eight-step reproducible pipeline, quality gates, the CORI operational cycle (Capture, Organise, Review, Inform), configurable risk-prioritisation and a prototype Ebola-specific module – is provided in a companion OSF preprint.^10^ The present communication summarises only what the case-application discussion requires and refers readers to the preprint for detail.

Pan-African Real-time Epidemiological Intelligence System consumes permitted inputs – WHO Disease Outbreak News and situation reports, Africa CDC bulletins, aggregate line lists and District Health Information Software 2 (DHIS2) exports where authorised and event-based signals where authorised – and produces provenance-tagged dashboards, prioritisation tables, risk maps and situation-summary templates. Current ingestion is from structured or semi-structured sources only; unstructured social-media crawling is on the roadmap and not yet operational.^10^ Any high or very high output requires qualified expert epidemiological review before being shared beyond the authorised team, and notification templates require explicit institutional authorisation prior to dissemination.^10^

Pan-African Real-time Epidemiological Intelligence System is not an autonomous open-source detection engine but an analytical intelligence layer that transforms already-captured, permitted signals into decision-oriented products. The distinction between detection capacity (identifying a signal from raw source data) and analytical capacity (transforming, contextualising and decision-orienting already-captured signals) is preserved throughout. Pan-African Real-time Epidemiological Intelligence System is designed to complement, not replace, established platforms including IDSR, EIOS, EWARS, ProMED, HealthMap and DHIS2, as summarised in Table 1; a fuller comparison appears under ‘Comparison with existing systems’ in the companion preprint.^10^

**TABLE 1:** Complementarity between the Pan-African Real-time Epidemiological Intelligence System proposed methodological framework and selected established epidemic-surveillance platforms in the African context.

Platform	Core function	Type of capacity	Coverage in African context	Complementary contribution proposed by PREIS
IDSR (WHO AFRO)	Integrated disease surveillance and response, including event-based surveillance in the third-edition guidelines.	Detection + notification	National and subnational; established regional framework.	May transform authorised IDSR-compatible aggregate outputs into reproducible dashboards, prioritisation tables and quality-control reports.
EIOS (WHO)	Open-source intelligence curation with regional community model and disease-specific board configurability.	Detection + curation	Global; regional teams maintain tailored context-specific sources.	May consume EIOS-derived signals as one of several permitted inputs, subject to permissions and add locally maintained analytical processing.
EWARS (WHO)	Early warning, alert and response support in emergency and humanitarian settings.	Detection + emergency response	Deployed in outbreak and humanitarian contexts.	May integrate permitted EWARS-type outputs with contextual and readiness data for reproducible cross-source analysis.
ProMED and HealthMap	Curated or automated Internet-based outbreak intelligence.	Detection	Global; open access.	May ingest permitted signals and situate them in configured regional classification schemas while preserving source-reliability flags.
Africa CDC EBS	Continental event-based surveillance framework.	Detection + coordination	Continental; institutional platform.	May structure downstream processing, deduplication, provenance tracking and reviewable outputs from authorised EBS signals.
DHIS2	Health information management platform for aggregate and event or tracker data.	Data management	Widely deployed in African Member States.	May consume authorised DHIS2 exports or APIs and link them to outbreak analytics and review workflows.

*Source*: Adapted from Zabre RH. PREIS: A modular, reproducible and AI-assisted framework for epidemic intelligence workflows in Africa [homepage on the Internet]. OSF Preprints; 2026 [cited 2026 July 04]. Available from: https://osf.io/ckzj4. Language deliberately conservative: Cells describe potential complementary contribution, not empirical performance benchmarking

PREIS, Pan-African Real-time Epidemiological Intelligence System; EWARS, Early Warning, Alert and Response System; IDSR, Integrated Disease Surveillance and Response; CDC, Centres for Disease Control and Prevention; WHO, World Health Organization; EIOS, Epidemic Intelligence from Open Sources; EBS, event-based surveillance; DHIS2, District Health Information Software 2; APIs, application programming interfaces; AFRO, World Health Organization Regional Office for Africa.

## Case application: The 2026 Bundibugyo virus disease Public Health Emergency of International Concern

The 2026 outbreak offers an operational case for examining how a PREIS-type analytical layer could contribute to signal integration in a live PHEIC context. It is used here as an illustrative case only: We do not report the causal effect of any intervention, present validated detection-performance estimates or reproduce restricted operational data. Any operational deployment would require formal institutional authorisation, written data-sharing agreements, expert-review protocols and communication safeguards consistent with international regulations.^10,11^

Four features of the 2026 outbreak inform the signal-integration discussion. Firstly, the initial alert (05 May) preceded laboratory confirmation (15 May) by 10 days.^1^ Secondly, the affected geography spanned three Ituri health zones (Bunia, Rwampara and Mongbwalu) with concurrent cross-border risk into Uganda.^1,2^ Thirdly, initial testing targeted the Zaire ebolavirus strain; identifying the Bundibugyo strain required specific sequencing, and no licensed Bundibugyo vaccine or therapeutic exists^2^ – an absence of post-detection countermeasures that amplifies the value of early multi-signal detection without itself constituting a detection-gap argument. Fourthly, the concurrent signals during the interval – syndromic surges, health-worker infections, cross-border mobility – were captured by existing platforms but not integrated into a single decision product at the required speed.^1^

Table 2 lists the principal signal types active during the outbreak and their relevance to interpretive integration. The framing is descriptive; no counterfactual claim is made about what a PREIS-type layer would have produced during the actual outbreak.

**TABLE 2:** Principal signal types active during the 2026 Bundibugyo virus disease outbreak context and their relevance to interpretive signal integration.

Signal type	Illustrative source	Relevance to the 2026 outbreak context
Syndromic surge in health facilities	Aggregate attendance data; facility spot reports; DHIS2 exports where authorised.	The 05 May 2026 WHO alert originated from Mongbwalu Health Zone, where syndromic patterns preceded laboratory confirmation by 10 days.^1^
Health-worker infections and deaths	Facility reports; district-level operational updates.	Deaths among health workers were noted in the initial 05 May 2026 WHO alert and served as a high-priority signal for outbreak declaration.^1^
Community mortality clustering	Event-based surveillance community reports; local health-authority notifications.	Community-level mortality signals in Ituri Province preceded formal laboratory confirmation; interpretation required community-signal integration.
Cross-border mobility signals	Border surveillance data; humanitarian mobility monitoring where authorised.	The DRC–Uganda corridor and the proximity of Bunia to the Uganda border generated cross-border risk documented in the WHO PHEIC determination.^2^
Open-source intelligence signals	ProMED-mail; local news feeds; EIOS-curated signals.	Open-source signals contributed to public awareness of the unfolding event; the interpretive-integration gap was not primarily a capture gap.^4,7,8^
Scientific and diagnostic intelligence	WHO technical notes; preprints; peer-reviewed literature.	Scientific intelligence on Bundibugyo virus characteristics and diagnostic tools became particularly important given the strain-specific nature of the outbreak.^2^

Note: Please see the full reference list of the article Zabré HR. Signal integration challenges in African epidemic intelligence: Lessons from the 2026 Bundibugyo virus disease outbreak and application of the PREIS framework. J Public Health Africa. 2026;17(1), a2079. https://doi.org/10.4102/jphia.v17i1.2079, for more information

PHEIC, Public Health Emergency of International Concern; EIOS, Epidemic Intelligence from Open Sources; DRC, Democratic Republic of the Congo; DHIS2, District Health Information Software 2; WHO, World Health Organization.

## Implications for African epidemic intelligence

The 2026 Bundibugyo virus disease PHEIC illustrates three lessons for African epidemic intelligence. Firstly, and most importantly, the principal challenge was not signal availability: Signals were present and existing platforms captured many of them; the gap lay in integrating concurrent multi-source signals into a unified decision product at the required speed. Secondly, established platforms – IDSR, EIOS, EWARS, ProMED, HealthMap, and DHIS2 – provide valuable, complementary capacities; their limitations are specific configuration and analytical-layer gaps amenable to complementary intervention rather than replacement. Thirdly, locally maintained analytical layers, developed by and for African public health practitioners under authorised data governance, may help strengthen interpretive-integration capacity – consistent with the Africa CDC blueprint for early warning surveillance^12^ and the priorities of Africa’s continental public health architecture.^13^

A framework such as PREIS should be understood as an adaptable analytical layer that may complement – not replace – established platforms.^14^ Its potential contribution is a reproducible workflow model that helps practitioners organise heterogeneous permitted signals into transparent, reviewable products. Realising this requires co-design with authorised institutions, formal data-sharing arrangements, prospective external validation against verified epidemiological ground truth and disease-specific peer-reviewed studies – none of which is complete at the time of writing, as the preprint makes explicit.^10^

## Governance, ethics, and institutional neutrality

Pan-African Real-time Epidemiological Intelligence System is presented as a proposed methodological and prototype framework. It is not, at the time of writing, an official platform, policy, product or endorsed tool of Africa CDC, the African Union, the World Health Organization (WHO), any Ministry of Health, any Regional Coordinating Centre or any Member State, unless future formal endorsement is obtained through appropriate institutional processes.^10^ References to Regional Coordinating Centres, IDSR, event-based surveillance (EBS), EIOS, EWARS, DHIS2 and other systems are made solely for methodological alignment, interoperability and comparative context; they imply no endorsement, affiliation, operational deployment, data ownership or official outbreak notification.

No individual-level human participant data are reported, and no confidential institutional dataset is reproduced; the case, death and geographic figures are drawn from publicly available WHO Disease Outbreak News and PHEIC determination statements, each cited with retrieval date. Analytical quality assurance is treated as an ethical requirement: In operational deployments, risk scores, novelty flags and notification templates should be reviewed by qualified epidemiologists before external sharing, and the framework should communicate uncertainty and distinguish official confirmed data from early attention signals.

## Conclusion

The 2026 Bundibugyo virus disease PHEIC illustrates that Africa’s principal detection challenge is often not signal availability but the timely interpretive integration of concurrent multi-source signals into unified decision products. Locally maintained, reproducible analytical layers that complement – rather than replace – established frameworks may help strengthen this capacity, subject to co-design with authorised institutions, formal data-governance arrangements and prospective validation.

Pan-African Real-time Epidemiological Intelligence System is proposed as one such analytical layer; its full methodology, prototype logic and explicit limitations are set out in the companion preprint.^10^ Future work should prioritise co-design with African public health institutions and competent authorities, formal data-governance arrangements, prospective external validation, implementation research and disease-specific peer-reviewed studies.
